# Supplementary material for: Neuron-level explainable AI for Alzheimer’s Disease assessment from fundus images
Source: Sci Rep. 2024 Apr 2;14:7710. doi: 10.1038/s41598-024-58121-8 (PMC10987553; doi:10.1038/s41598-024-58121-8)
Supplement: Supplementary file 1 — Supplementary Information. [file 41598_2024_58121_MOESM1_ESM.pdf]

Supplementary Information

A Supplementary Methods

Adjacency-constrained Hierarchical Agglomerative Clustering. Hierarchical clustering can be generated either top-down called *divisive clustering* similar to k-means (where a data set is divided into more number of smaller clusters gradually) or bottom-up called *agglomerative clustering* (where initially every data point is considered as an individual cluster and then gradually merged into less number of bigger clusters). Divisive clustering can be linear in the number of clusters if the number of top levels is fixed, despite that the number of clusters in LAVA formulation is not pre-defined and depends on the application and the granularity nature of the data structure. We use Hierarchical Agglomerative Clustering (HAC) with the ward's linkage. The time complexity of naive agglomerative clustering is $O(n^3)$ and can be reduced to $O(n^2 logn)$ when priority queue data structure is used and can be reduced to $O(n^2)$ with some further optimization. In the HAC algorithm, the between-cluster agglomerative distance can be recursively computed, while the aggregated distance between clusters can be updated without the need to compute all the pairs of objects contained in the clusters. In this setting, we use the ward's linkage to update the aggregated distance between clusters. This approach attempts to merge two clusters for which the change in total variation is minimized. The total variation of a clustering result is the sum of squared-error $ESS(C)$ (so-called *inertia of cluster C* [**?**]) between every object and the centroid of the cluster containing that object. Thus, Ward's linkage criterion δ can be formulated as follows when two clusters C and C' are merged where $\bar{C}$ is the mean vector (centroid) of the clusters.

$$\delta(C, C') = ESS(C \cup C') - ESS(C) - ESS(C') \tag{A.1}$$

$$ESS(C) := \frac{1}{|C|} \sum_{i \in C} ||x_i - \bar{C}||^2 \tag{A.2}$$

Suppose we have two clusters C and C' that are merged into a new cluster C^*, and let C'' be any other cluster. Let the size of cluster C , C' , C'' be n_c, n_c', n_c'' correspondingly. Algorithm updates distance $D(C^*, C'')$ from $D(C, C'')$ and $D(C', C'')$ through a systematic pairwise distance $D(x_i, x_j)$ for every $i \neq j$ is given as follows.

$$\begin{aligned} D(C^*, C'') = \frac{n_c + n_c''}{n_c + n_c' + n_c''} D(C, C'') + \\ \frac{n_c' + n_c''}{n_c + n_c' + n_c''} D(C', C'') - \frac{n_c''}{n_c + n_c' + n_c''} D(C, C') \end{aligned} \tag{A.3}$$

In our experiment, we use the entire set of input images for which the array of features (critical neurons' activation) serves as raw training data so-called

activation dataset in the constrained version of the clustering algorithm in a semi-supervised setting.

Given k different parameterization of a classifier model Φ through nested k-fold cross-validation learning paradigm, with L' selected layers where $l' = \{1, .., L'\}$, and $N = \{1, ..., N\}$ total number of input samples $X = \{x_1, ..., x_n\}$. let $\{Z^k_{l'}\}_{i=1}^N$ denotes the activation of critical neurons at selected layer l' of k-th model for i-th input instance. To aggregate activation values, first, we stack activation values across all cross-validating models $\{Z_{l'}\}_{i=1}^N$. Second, we stack them across all selected layers $\{Z\}_{i=1}^N$ (where i is an index of input sample instance) to construct a two-dimensional array of the activation values of critical neurons across entire networks over all input samples.

Let $Y = \{y_1, ...y_n\}$ denote the array of ground-truth labels for all input images. We construct the connectivity graph $h = \{0, 1\}^{N\times N}$ out of the k-nearest neighbor graph (K-NNG) as constraints in a semi-supervised learning algorithm. In this graph, if the distance between two nodes p and q is among the kth smallest distance from node p to any other nodes, p and q are connected. In this setting, the standard Euclidean metric measures the difference of ground truth labels assigned to each sample point. The output of this algorithm is a sparse CSR-format connectivity matrix A of shape $N \times N$ where only $k \times N$ number of entries (self-included) are one and the rest are zero. This algorithm reduces a chunk of distances to the k-nearest neighbors where elements are partitioned by element index $k - 1$ in the stable sorted array of distances for each sample instance.

Connectivity constraints make the clustering algorithm perform differently in the constrained version of HAC in two aspects:

1. After each step of merging, a graph $h^{(p)}$ will be created (recursively) to record the connectivity constraints between clusters at any iteration p where current clusters are treated as nodes in the graph.
2. Two clusters can be merged only if they are connected according to the connectivity constraint graph at the current iteration $h^{(p)}$.

The pseudocode of this clustering method is provided in Supplementary Algorithm 2.

B Supplementary Algorithms

Algorithm 1 LAVA - Neuron-level Probing

Input:
A binary vector $\hat{Y} = (\hat{y}_1, ..\hat{y}_n)$ of predicted labels for n input samples $X = (x_1, ..x_n)$.
A set of all neurons at L layers denoted as $\{S_l\}_{l=1}^{L}$ and their activation values for all input samples, denoted as $\{Z_l\}_{l=1}^{L}$.
A positive integer P number of critical neurons to extract from the selected layer.
A Kernel type.
An equal or greater than 1 integer for regularization parameter C.

Output:
Set of critical neurons at each layer $\{\hat{S}_l\}_{l=1}^{L}$ and their activation values $\{Z'_l\}_{l=1}^{K}$.

for $l = 1$ to L **do** ▷ at each selected layer
    scores $\Leftarrow$ coefficient of the contribution of neurons to the output of the model $\hat{Y}$ estimated by ϵ-SVR on $\{Z_l\}_{l=1}^{L}$.
    $\hat{S}_l \Leftarrow$ recursively eliminate the least important neurons based on the *scores* until P is reached by RFE.
    $Z'_l \Leftarrow$ filter activation values for only critical neurons at each layer.
end for
return $\hat{S}$ and Z'.

Algorithm 2 LAVA - Granularity Explanation

Input:
An array Z' of activation values of critical neurons at L layers for all samples (obtained from Algorithm 1)
A positive integer K number of nearest neighbors.
A binary vector $Y = (y_1, ..y_n)$ of target labels for n input samples $X = (x_1, ..x_n)$.
Ward's linkage criterion δ.
A pairwise distance metric ζ.
A positive integer R number of clusters.

Output :
A ward tree representation U of input samples.
A vector of cluster labels W for input samples.

Linkage matrix construction
$h = \{0, 1\}^{n \times n} \Leftarrow$ construct sparse connectivity matrix with Y and K
Semi-supervised learning
Construct distance matrix M on Z' with ζ.
$(C^0) = (C_i^0)_{1 \leq i \leq N}$ with $C_i^0 = \{x_i\}$ ▷ initializing clusters
Initialize $h^{(0)}$ graph on clusters C_i^0 as vertices.
for $i = 1$ to $n - 1$
if $(C_u^{(i-1)}, C_{u+1}^{(i-1)}) \in h^{(i-1)}$ ▷ find best merging candidate
$d_t = argmin_{d \in \{1,...,N-i\}} \delta(C_u^{i-1}, C_{u+1}^{i-1})$
Update graph $h^{(i)}$ on new clusters.
Update matrix M by removing row and column of merged clusters.
for $d = 1$ to $n - i - 1$ ▷ update C^i with C^{i-1}
if $d < d_i$ then $C_u^i = C_u^{i-1}$
else if $d = d_i$ then $C_u^i = C_u^{i-1} \cup C_{u+1}^{i-1}$
else if $d > d_i$ then $C_u^i = C_{u+1}^{i-1}$
end if
end For
end for
$g \Leftarrow$ constructed hierarchical ward tree
Fit g on Z' for R clusters.
$U \Leftarrow$ transformation of Z' by ward tree g
$W \Leftarrow$ predict sub-class labels on Z' by ward tree g
return U and W.

C Supplementary Figures

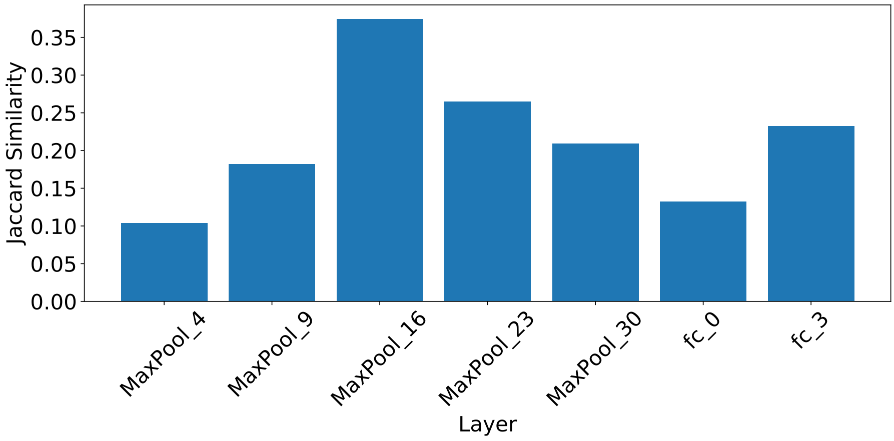

Supplementary Figure 1: **Critical neurons identification.** Overlapping between sets of critical neurons at different layers of the network identified repetitively by different parameterizations of the model obtained from K-fold cross-validation is measured by Jaccard similarity.

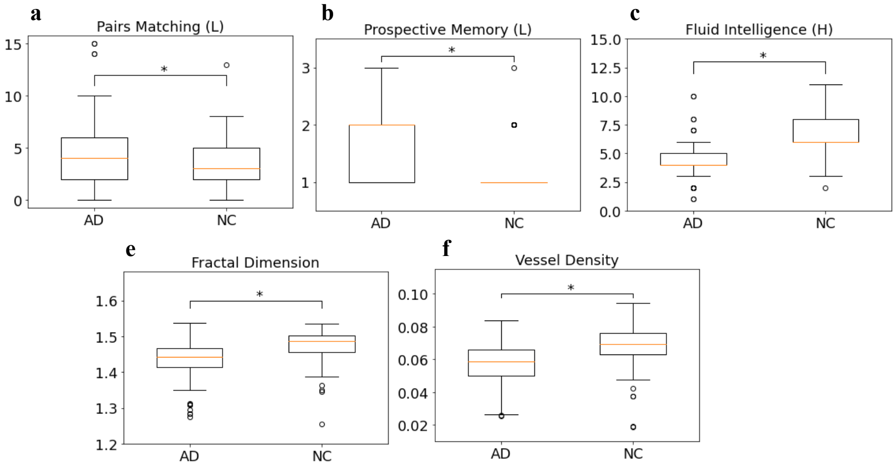

Supplementary Figure 2: **Box-plot comparisons between AD and NC groups of cognitive and vascular features.** (*) indicates statistical significance ($p < 0.01$) by two-tailed significance tests.

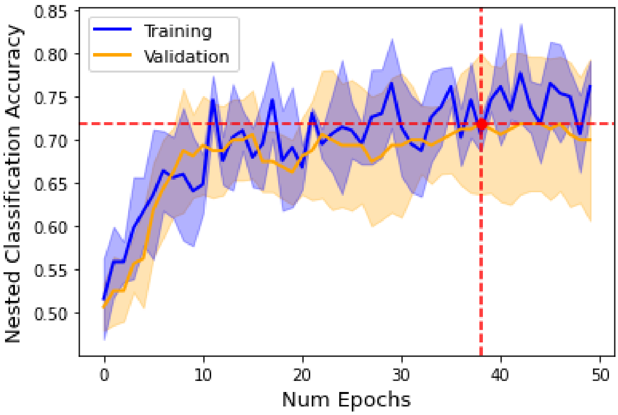

Supplementary Figure 3: **Training and Validation Performances during the Nested Cross Validation**. In bold lines are the average classification accuracy during training and validation, and the shaded area represents the standard deviation over the nested-cross-validation. The optimal number of epochs is indicated by the red dashed lines (early stopping). After the optimal parameters are found, the model is re-trained by reconsolidating all of the training data to leverage the minimal data maximally.

D Supplementary Tables

	CN-1	CN-2	CN-3	Mixed	AD-1	AD-2	AD-3	Total
AD	0	0	0	31	21	30	18	100
CN	15	39	15	31	0	0	0	100

Supplementary Table 1: **Results of partitioning data samples by LAVA.** In this study, the granularity level is configured to diagnose 7 subgroups of subjects corresponding to different stages of AD progression. In total 3 AD groups, 3 CN groups and 1 Mixed group are identified by the LAVA framework.

Baseline Characteristics	AD Group	HC Group	pvalue
N	61	80	–
Age, mean (SD), years	64.5 (3.6)	63.9 (4.1)	0.35
Gender, No. (%)			0.94
Male	27 (44)	34 (45)	
Female	34 (56)	44 (55)	
Ethnicity, No. (%)			0.74
White	56 (91.8)	78 (97.5)	
Others	5 (8.2)	2 (2.5)	
Eye Problems (%)			0.98
Yes	10 (16.4)	13 (16.3)	
No	51 (83.6)	67 (83.8)	
Visual Acuity, mean (SD), LogMAR	0.11 (0.20)	0.06 (0.20)	0.16
Townsend Indices, mean (SD)	-1.43 (3.30)	-1.55 (2.61)	0.81
Diabetes-Obesity, No. (%)			0.19
Yes	18 (29.5)	16 (20)	
No	43 (70.49)	64 (80)	
Smoking Status, No. (%)			0.13
Yes	33 (54.1)	33 (41.3)	
No	28 (45.9)	47 (58.8)	
Alcohol Status, No. (%)			0.18
Yes	60 (98.4)	75 (93.8)	
No	1 (1.6)	5 (6.3)	
History of Stroke, No. (%)			0.01*
Yes	8 (13.1)	2 (2.5)	
No	53 (86.9)	78 (97.5)	

Supplementary Table 2: **Baseline characteristics of the study populations**. P-values conducted on continuous data are computed by the Student's t-test. Categorical variables are computed by Pearson's Chi-squared test. * indicates statistically significant ($p < 0.05$).
